# Incremental Predictive Value of the Non-HDL-C/HDL-C Ratio for Cryptogenic Stroke in Patients with Patent Foramen Ovale

**DOI:** 10.3390/jcdd13070313

**Published:** 2026-07-08

**Authors:** Tarik Yildirim, Mehmet Tolga Hekim, Tuncay Kiris

**Affiliations:** 1 Department of Cardiology, School of Medicine, Balikesir University, Balikesir 10145, Turkey; 2 Department of Cardiology, Izmir Katip Çelebi University, Atatürk Training and Research Hospital, Izmir 35360, Turkey

**Keywords:** patent foramen ovale, cryptogenic stroke, non-HDL-C/HDL-C ratio, ROPE score, risk stratification

## Abstract

**Background**: The non-high-density lipoprotein cholesterol (non-HDL-C) / high-density lipoprotein cholesterol (HDL-C) ratio has emerged as a marker of residual vascular risk; however, its role in patent foramen ovale (PFO)-associated cryptogenic stroke (CS) remains unclear. We investigated the association between the non-HDL-C/HDL-C ratio and CS in patients with PFO and evaluated its incremental predictive value beyond the Risk of Paradoxical Embolism (ROPE) score. **Methods**: This retrospective study included 316 patients with confirmed PFO, including 56 patients with CS. Multivariable logistic regression, restricted cubic spline analysis, ROC analysis, net reclassification improvement (NRI), integrated discrimination improvement (IDI), decision curve analysis, and bootstrap internal validation were performed. **Results**: Patients with CS had significantly higher non-HDL-C/HDL-C ratio levels than those without CS (*p* < 0.001). In multivariable analysis, the non-HDL-C/HDL-C ratio remained independently associated with CS (OR: 1.881, 95% CI: 1.310–2.700, *p* < 0.001). Restricted cubic spline analysis demonstrated a significant nonlinear association between the non-HDL-C/HDL-C ratio and CS risk (overall *p* = 0.001; nonlinear *p* = 0.015). Addition of the non-HDL-C/HDL-C ratio to the ROPE score improved discrimination, increasing the AUC from 0.781 to 0.819 (DeLong *p* = 0.010), and significantly improved risk reclassification (continuous NRI: 0.555, *p* = 0.002; IDI: 0.057, *p* = 0.002). Internal validation demonstrated stable model performance with minimal optimism. **Conclusions**: The non-HDL-C/HDL-C ratio was independently associated with CS and demonstrated potential incremental predictive value beyond the ROPE score in patients with PFO. These findings suggest that metabolic lipid burden may contribute to thromboembolic susceptibility and may improve individualized risk stratification in PFO-related stroke.

## 1. Introduction

Patent Foramen Ovale (PFO) is present in approximately 20–25% of the general population and is observed more frequently among patients with cryptogenic stroke (CS) [1,2]. Although paradoxical embolism is considered the principal mechanism linking PFO to ischemic events, most individuals with PFO remain asymptomatic, suggesting that additional risk modifiers contribute to thromboembolic susceptibility [3].

The Risk of Paradoxical Embolism (ROPE) score was developed to estimate the likelihood that a detected PFO is causally related to stroke [4]. More recently, contemporary reviews have emphasized that risk assessment in patients with PFO should integrate clinical, anatomical, and patient-specific factors to better identify pathogenic PFO and guide individualized management [5]. However, the ROPE score primarily incorporates age and conventional vascular risk factors and does not account for metabolic determinants of thrombosis. Dyslipidemia has been implicated in endothelial dysfunction, platelet activation, and systemic prothrombotic states, mechanisms that may increase embolic susceptibility in patients with PFO beyond its established role in atherosclerosis [6,7].

Non-high-density lipoprotein cholesterol (non-HDL-C) reflects the total burden of atherogenic lipoproteins, while HDL cholesterol (HDL-C) exerts anti-inflammatory and antithrombotic effects. The non-HDL-C/HDL-C ratio integrates both components and has emerged as a strong predictor of adverse cardiovascular outcomes [8,9]. Nevertheless, its potential role in PFO-associated CS has not been adequately investigated.

Therefore, we aimed to evaluate the association between the non-HDL-C/HDL-C ratio and CS in patients with PFO and to determine whether this metabolic index provides incremental discriminatory value beyond the ROPE score.

## 2. Methods

This retrospective observational study included 316 consecutive patients with confirmed Patent Foramen Ovale evaluated between January 2018 and December 2025 at a tertiary cardiovascular center. PFO diagnosis was established by transesophageal echocardiography with contrast-enhanced bubble study. Patients were classified according to the presence of CS: 56 patients (17.7%) constituted the CS group and 260 patients (82.3%) had no history of CS. CS was defined as an ischemic cerebrovascular event confirmed by neuroimaging in the absence of an alternative identifiable etiology after standard diagnostic work-up, including cardiac rhythm monitoring and vascular imaging. In patients with cryptogenic stroke, atrial fibrillation was excluded by standard diagnostic evaluation, including 12-lead electrocardiography during hospitalization and 24 h Holter ECG monitoring. Patients with atrial fibrillation, significant carotid stenosis (≥50%), known non-PFO cardioembolic sources, active malignancy, or incomplete or missing data were excluded. The study protocol complied with the Declaration of Helsinki and was approved by the institutional ethics committee.

Baseline demographic and clinical variables included age, sex, hypertension, diabetes mellitus, hyperlipidemia, smoking status, and chronic obstructive pulmonary disease. Echocardiographic parameters comprised left ventricular ejection fraction, presence of interatrial septal aneurysm, PFO tunnel length and diameter (mm), and Eustachian valve. The ROPE score was calculated for all patients using the original ROPE score described by Kent et al. [4].

Fasting blood samples were obtained at admission and included hemoglobin, platelet count, leukocyte count, glucose, creatinine, total cholesterol, LDL-C, HDL-C, and triglycerides. Non-HDL-C was calculated as total cholesterol minus HDL-C. The primary metabolic variable was the non-HDL-C/HDL-C ratio.

### Statistical Analysis

Continuous variables were tested for normality using the Kolmogorov–Smirnov test and are presented as mean ± standard deviation or median (interquartile range), as appropriate. Group comparisons were performed using the independent samples *t*-test or Mann–Whitney U test. Categorical variables were compared using the chi-square or Fisher’s exact test.

Covariates included in the multivariable models were selected a priori based on clinical relevance and evidence from previous studies, including the ROPE score, glucose level, PFO tunnel length, PFO tunnel diameter, and the non-HDL-C/HDL-C ratio. Results are presented as odds ratios (ORs) with 95% confidence intervals (CIs).

Restricted cubic spline analysis was performed using the rms package in R to flexibly evaluate the association between the non-HDL-C/HDL-C ratio and the probability of cryptogenic stroke without assuming a linear relationship. The spline model was fitted using three knots, with knot locations automatically determined by the default algorithm implemented in the rms package according to the distribution of the predictor variable. Nonlinearity was assessed using a Wald test for the nonlinear spline terms. In addition, patients were categorized according to quartiles of the non-HDL-C/HDL-C ratio, and multivariable logistic regression analyses were conducted using the lowest quartile as the reference category.

To assess the incremental predictive value of the non-HDL-C/HDL-C ratio, two predictive models were constructed: the ROPE score alone and the ROPE score combined with the non-HDL-C/HDL-C ratio. Discriminatory performance was evaluated using the area under the receiver operating characteristic curve (AUC), and differences between correlated ROC curves were compared using DeLong’s test. Continuous net reclassification improvement (NRI) and integrated discrimination improvement (IDI) were also calculated.

Internal validation was performed using bootstrap resampling (1000 iterations). Model calibration was assessed using calibration plots, calibration slope and intercept, Brier score, and the Hosmer–Lemeshow goodness-of-fit test. Clinical utility of the prediction models was further evaluated using decision curve analysis. A two-sided *p* value < 0.05 was considered statistically significant. All statistical analyses were performed using SPSS software version 30.0 (IBM Corp., Armonk, NY, USA), and R software version 4.5.1 (R Foundation for Statistical Computing, Vienna, Austria).

## 3. Results

### 3.1. Baseline Clinical Characteristics and Echocardiographic Findings

A total of 316 patients were included in the study, comprising 260 patients without CS and 56 patients with CS. Patients with CS were significantly older than those without CS (51.9 ± 11.4 vs. 45.8 ± 12.6 years, *p* = 0.001, Table 1). Hypertension (46.4% vs. 30.4%, *p* = 0.023), diabetes mellitus (21.4% vs. 10.4%, *p* = 0.028), hyperlipidemia (41.1% vs. 22.3%, *p* = 0.004), and coronary artery disease (19.6% vs. 7.3%, *p* = 0.006) were more frequent in patients with CS. In addition, interatrial septal aneurysm (42.9% vs. 27.7%, *p* = 0.026) and the presence of a Eustachian valve (25.0% vs. 13.5%, *p* = 0.031, Table 1) were significantly more common in the CS group. Patients with stroke also had significantly greater PFO tunnel length (11.4 ± 4.1 vs. 9.1 ± 3.8 mm, *p* = 0.001) and tunnel diameter (3.2 ± 1.0 vs. 2.7 ± 0.9 mm, *p* = 0.002, Table 1).

### 3.2. Laboratory Findings

Patients with CS demonstrated significantly higher glucose levels [110 (96–136) vs. 102 (92–118) mg/dL, *p* = 0.041, Table 1], total cholesterol levels (211 ± 43 vs. 192 ± 39 mg/dL, *p* = 0.002), LDL-C levels (132 ± 36 vs. 118 ± 33 mg/dL, *p* = 0.010), triglyceride levels [172 (121–226) vs. 146 (104–192) mg/dL, *p* = 0.012], and non-HDL cholesterol levels (170 ± 40 vs. 146 ± 37 mg/dL, *p* < 0.001). HDL-C levels were significantly lower in the CS group (41 ± 10 vs. 46 ± 11 mg/dL, *p* = 0.003). The non-HDL-C/HDL-C ratio was markedly elevated in patients with CS [3.56 (2.82–4.42) vs. 2.86 (2.30–3.45), *p* < 0.001, Table 1]. Furthermore, ROPE scores differed significantly between groups (5.8 ± 1.6 vs. 6.4 ± 1.7, *p* = 0.018, Table 1).

### 3.3. Multivariable Analysis

In multivariable analysis, the non-HDL-C/HDL-C ratio remained an independent predictor of CS (OR: 1.881, 95% CI: 1.310–2.700, *p* < 0.001), together with ROPE score (OR: 1.848, 95% CI: 1.490–2.295, *p* < 0.001), glucose level (OR: 1.016, 95% CI: 1.002–1.031, *p* = 0.030), PFO tunnel length (OR: 1.096, 95% CI: 1.003–1.196, *p* = 0.042), and PFO tunnel diameter (OR: 1.425, 95% CI: 1.063–1.911, *p* = 0.018, Table 2).

**Figure 1:**
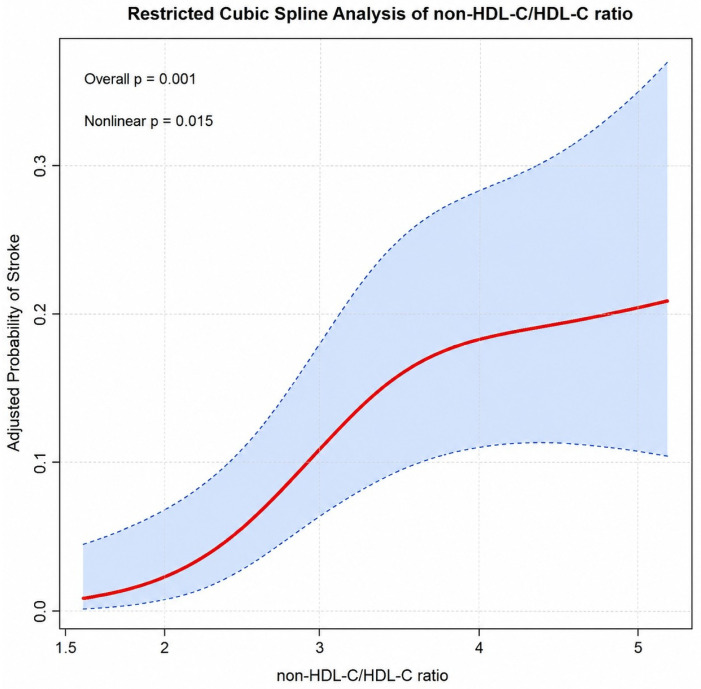
Restricted cubic spline analysis demonstrating the association between non-HDL-C/HDL-C ratio and adjusted probability of stroke. The red solid line represents the adjusted predicted probability of stroke, while the shaded blue area and dashed lines indicate the 95% confidence interval.

Restricted cubic spline analysis demonstrated a significant nonlinear association between the non-HDL-C/HDL-C ratio and CS risk (overall *p* = 0.001; nonlinear *p* = 0.015, Figure 1), with progressively increasing adjusted CS probability at higher non-HDL-C/HDL-C ratio values. Quartile analysis further confirmed a dose–response relationship. Compared with the lowest quartile, patients in the second, third, and fourth quartiles had significantly increased adjusted odds of CS, with ORs of 3.95 (95% CI: 1.13–17.13, *p* = 0.033), 5.95 (95% CI: 1.75–25.63, *p* = 0.004), and 9.41 (95% CI: 2.77–40.79, *p* < 0.001, Table 3), respectively. A significant positive trend in CS risk was observed across increasing quartiles of the non-HDL-C/HDL-C ratio (*p* for trend < 0.001).

### 3.4. Incremental Predictive Value

**Figure 2:**
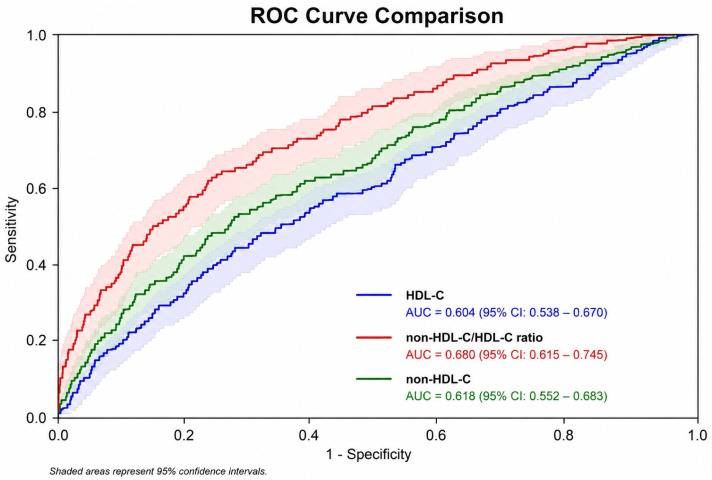
Receiver operating characteristic (ROC) curve comparison of HDL-C, non-HDL-C, and non-HDL-C/HDL-C ratio for prediction of stroke.

Receiver operating characteristic analysis showed that the non-HDL-C/HDL-C ratio had a higher AUC than HDL-C (0.680 vs. 0.604, *p* = 0.039) and non-HDL-C (0.680 vs. 0.618, *p* = 0.035), whereas HDL-C and non-HDL-C had comparable discriminatory performance (*p* = 0.809; Figure 2).

**Figure 3:**
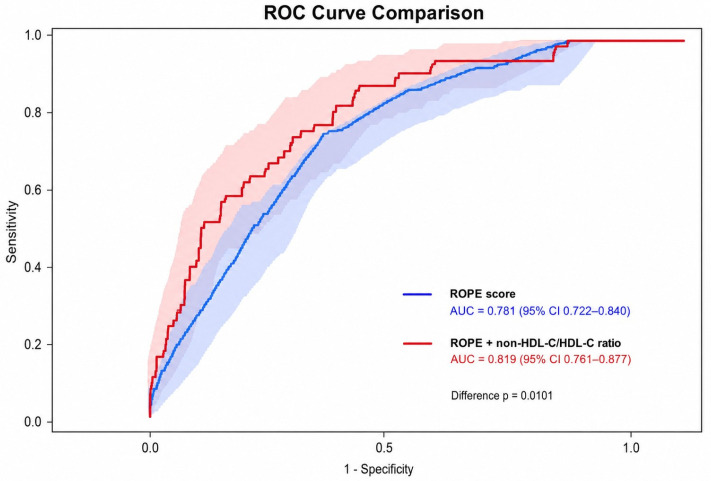
ROC curve comparison between the ROPE score alone and the combined model including ROPE score plus non-HDL-C/HDL-C ratio for prediction of stroke. The shaded areas indicate the 95% confidence intervals of the variables.

**Figure 4:**
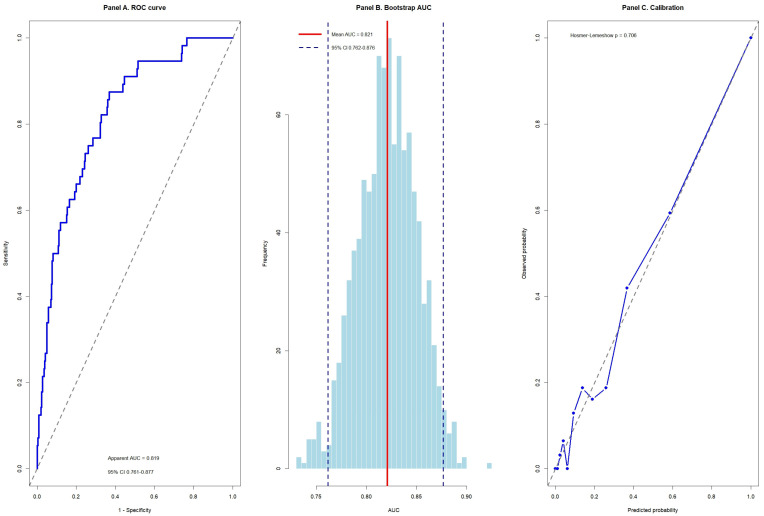
Internal validation and calibration analysis of the combined prediction model. Panel (**A**) demonstrates the ROC curve of the combined model with an apparent AUC of 0.819 (95% CI 0.761–0.877). Panel (**B**) shows bootstrap validation results, demonstrating stable model performance with a mean bootstrap AUC of 0.821 and 95% CI of 0.762–0.876. Panel (**C**) presents the calibration plot, demonstrating good agreement between predicted and observed stroke probabilities (Hosmer–Lemeshow *p* = 0.706).

To evaluate the incremental predictive value beyond the ROPE score, a combined model incorporating both the ROPE score and the non-HDL-C/HDL-C ratio was constructed. The combined model demonstrated a modest but statistically significant improvement in discrimination, with the AUC increasing from 0.781 (95% CI: 0.722–0.844) to 0.819 (95% CI: 0.761–0.877) (ΔAUC = 0.038; DeLong z = 2.573, *p* = 0.010; Figure 3). Addition of the non-HDL-C/HDL-C ratio also significantly improved risk reclassification, with a continuous NRI of 0.555 (95% CI: 0.235–0.859, *p* = 0.002) and an IDI of 0.057 (95% CI: 0.016–0.118, *p* = 0.002). Decision curve analysis further demonstrated greater net clinical benefit across a broad range of clinically relevant threshold probabilities (Figure 4).

**Figure 5:**
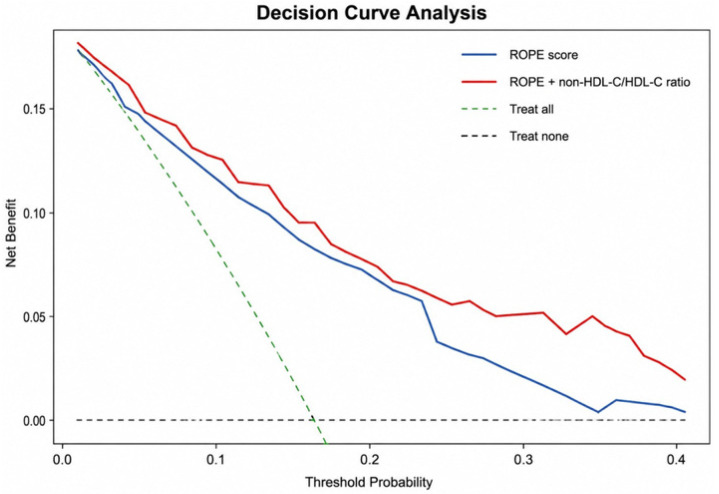
Decision curve analysis comparing the clinical utility of the ROPE score alone and the combined model including ROPE score plus non-HDL-C/HDL-C ratio. Across a wide range of threshold probabilities, the combined model provided greater net clinical benefit than the ROPE score alone, as well as the “treat all” and “treat none” strategies.

Internal validation using 1000 bootstrap resamples confirmed the stability of the combined model, yielding a mean bootstrap AUC of 0.821 (95% CI: 0.762–0.876). Calibration analysis demonstrated excellent agreement between predicted and observed CS probabilities, with a calibration slope of 1.00, calibration intercept close to zero, Brier score of 0.114, and no evidence of poor fit according to the Hosmer–Lemeshow test (*p* = 0.706, Figure 5). Bootstrap validation further demonstrated minimal model optimism, with an optimism-corrected AUC of 0.817 compared with an apparent AUC of 0.819, while calibration-in-the-large was approximately zero and the shrinkage factor was 1.00, indicating minimal evidence of overfitting.

## 4. Discussion

In this study, we demonstrated that the non-HDL-C/HDL-C ratio was independently associated with cryptogenic stroke in patients with patent foramen ovale. Moreover, incorporation of the non-HDL-C/HDL-C ratio into the ROPE score significantly improved discriminatory performance and risk stratification. These findings suggest that metabolic lipid burden may contribute additional prognostic information beyond conventional clinical and anatomical predictors, as summarized in the Central Illustration.

Although PFO is present in approximately one-quarter of the general population, only a subset of individuals experience paradoxical embolic events, indicating that additional modifiers influence thromboembolic susceptibility [1,10]. The ROPE score was developed to distinguish pathogenic from incidental PFOs and remains one of the most widely used tools for estimating the probability that a detected PFO is causally related to stroke [4,11]. A recent comprehensive review by Kent and Wang emphasized that contemporary assessment of PFO-associated stroke should combine clinical probability, anatomical characteristics, and individualized risk assessment, rather than relying on a single parameter [5]. However, the ROPE score primarily incorporates demographic and conventional vascular risk variables and does not account for systemic metabolic or inflammatory factors that may contribute to thrombogenesis.

In recent years, increasing attention has focused on the role of non-traditional lipid parameters in cardiovascular and cerebrovascular disease. Non-HDL-C reflects the cumulative burden of all atherogenic lipoproteins, including LDL particles, very-low-density lipoproteins (VLDL), intermediate-density lipoproteins, remnant lipoproteins, and lipoprotein(a), all of which may contribute to endothelial dysfunction, vascular inflammation, and thrombosis [6,7,12]. In contrast, HDL-C exerts anti-inflammatory, antioxidative, endothelial-protective, and antithrombotic effects [13,14,15,16]. Therefore, the non-HDL-C/HDL-C ratio integrates both atherogenic burden and protective lipid capacity into a single parameter and may more comprehensively reflect vascular vulnerability than isolated lipid indices.

Our findings are consistent with accumulating evidence suggesting that non-traditional lipid indices provide superior prognostic information compared with conventional lipid measurements alone [6]. Previous studies demonstrated that elevated non-HDL-C and non-HDL-C/HDL-C ratio levels are associated with recurrent stroke, adverse vascular outcomes, and residual cardiovascular risk despite adequate LDL-C control [6,7]. In particular, Liu et al. reported that elevated non-HDL-C/HDL-C ratio significantly increased recurrent stroke risk among elderly patients [8], whereas Kim et al. demonstrated that non-traditional lipid indices predicted unfavorable vascular outcomes even in statin-treated patients with controlled LDL-C levels [9]. Our study extends these observations to the specific population of PFO-associated CS and suggests that systemic metabolic lipid burden may interact with paradoxical embolic mechanisms.

Several biological mechanisms may explain the observed association between the non-HDL-C/HDL-C ratio and CS. Dyslipidemia promotes endothelial dysfunction, oxidative stress, platelet activation, thrombin generation, and inflammatory signaling pathways, all of which facilitate thrombus formation [13,14,15,16,17]. Elevated non-HDL-C levels reflect increased concentrations of highly atherogenic remnant particles that possess pro-inflammatory and pro-thrombotic properties [12,17]. Conversely, reduced HDL-C levels impair reverse cholesterol transport and diminish the anti-inflammatory and antithrombotic functions of HDL particles [14,15]. In the presence of a right-to-left shunt such as PFO, this prothrombotic milieu may increase the likelihood that venous microthrombi bypass pulmonary filtration and result in cerebral embolization [1]. The observed association may reflect a systemic prothrombotic and endothelial-vulnerable phenotype in which atherogenic lipoprotein burden facilitates thrombus propagation and embolic susceptibility in the presence of a right-to-left shunt.

Importantly, our results support a multifactorial model of PFO-related stroke in which both anatomical and systemic factors jointly contribute to embolic risk [4,18,19]. In our cohort, PFO tunnel length and tunnel diameter remained independent predictors of CS together with the non-HDL-C/HDL-C ratio and ROPE score. Furthermore, restricted cubic spline analysis demonstrated a significant nonlinear association between the non-HDL-C/HDL-C ratio and stroke probability, while quartile analysis revealed a clear dose–response relationship, with nearly tenfold higher adjusted odds of CS in the highest quartile compared with the lowest quartile. These findings suggest that increasing metabolic lipid burden progressively amplifies thromboembolic susceptibility in patients with PFO. Although patients with cryptogenic stroke exhibited a greater burden of conventional cardiovascular risk factors, the association between the non-HDL-C/HDL-C ratio and stroke remained significant after adjustment for clinically relevant covariates. Nevertheless, residual confounding inherent to observational studies cannot be completely excluded. Therefore, the observed association may reflect both systemic metabolic vulnerability and the overall cardiovascular risk profile of patients with cryptogenic stroke.

Beyond demonstrating an independent association, addition of the non-HDL-C/HDL-C ratio to the ROPE score resulted in modest but statistically significant improvements in discrimination and risk reclassification, while decision curve analysis demonstrated superior net clinical benefit across clinically relevant threshold probabilities. Together, these findings suggest that integrating metabolic lipid indices with established clinical scores may improve identification of high-risk patients with PFO [3,11,20,21].

The clinical implications of our findings are potentially important. Current risk stratification strategies in PFO primarily focus on anatomical features and conventional vascular risk factors [3,4,22]. However, our results suggest that systemic metabolic susceptibility may also play a relevant role in determining embolic risk. Identification of patients with elevated non-HDL-C/HDL-C ratio may have the potential to refine individualized risk assessment if confirmed in prospective studies. However, the present findings should be considered hypothesis-generating, and prospective multicenter studies with external validation are required before the non-HDL-C/HDL-C ratio can be considered for routine clinical risk stratification in patients with PFO.

A major strength of the present study is its comprehensive analytical approach. In addition to multivariable logistic regression, we evaluated the dose–response relationship using restricted cubic spline analysis and assessed the incremental predictive value of the non-HDL-C/HDL-C ratio using ROC analysis, NRI, IDI, and decision curve analysis. Furthermore, internal validation using bootstrap resampling demonstrated stable model performance, while calibration analyses showed excellent agreement between predicted and observed probabilities, with favorable calibration slope, intercept, Brier score, and Hosmer–Lemeshow statistics. These findings support the robustness and reliability of the proposed prediction model.

Several limitations should be acknowledged. First, this was a retrospective single-center study and therefore subject to inherent selection bias. Second, lipid measurements were obtained at a single time point and may not fully reflect long-term metabolic exposure. Third, despite comprehensive adjustment, residual confounding cannot be excluded. Fourth, because of the observational design, causality between elevated non-HDL-C/HDL-C ratio and CS cannot be definitively established. Fifth, the relatively limited number of events may have increased the risk of model overfitting despite internal validation procedures. Sixth, Sixth, although standard rhythm evaluation, including 12-lead electrocardiography and 24 h Holter ECG monitoring, was performed in patients with cryptogenic stroke, prolonged rhythm monitoring was not routinely available. Therefore, occult paroxysmal atrial fibrillation cannot be completely excluded. Finally, the findings of the present study should be regarded as hypothesis-generating until confirmed in prospective multicenter studies with external validation.

## 5. Conclusions

In patients with patent foramen ovale, the non-HDL-C/HDL-C ratio was independently associated with cryptogenic stroke and demonstrated potential incremental predictive value beyond the ROPE score. These findings should be considered hypothesis-generating and suggest that metabolic lipid burden may contribute to thromboembolic susceptibility in PFO-related stroke. Prospective multicenter studies with external validation are required before the non-HDL-C/HDL-C ratio can be considered for routine clinical risk stratification in patients with PFO.

## Figures and Tables

**Table 1:** Clinical Characteristics, Laboratory Parameters and Echocardiographic findings according to cryptogenic stroke.

Variable	Cryptogenic Stroke (−) N = 260	Cryptogenic Stroke (+) N = 56	*p* Value
Age (years)	45.8 ± 12.6	51.9 ± 11.4	0.001
Male gender, n (%)	148 (56.9%)	35 (62.5%)	0.44
Hypertension, n (%)	79 (30.4%)	26 (46.4%)	0.023
Diabetes mellitus, n (%)	27 (10.4%)	12 (21.4%)	0.028
Hyperlipidemia, n (%)	58 (22.3%)	23 (41.1%)	0.004
Coronary artery disease, n (%)	19 (7.3%)	11 (19.6%)	0.006
Smoking, n (%)	88 (33.8%)	26 (46.4%)	0.09
COPD, n (%)	9 (3.5%)	4 (7.1%)	0.18
Interatrial septal aneurysm, n (%)	72 (27.7%)	24 (42.9%)	0.026
Eustachian valve present, n (%)	35 (13.5%)	14 (25.0%)	0.031
Tunnel length (mm)	9.1 ± 3.8	11.4 ± 4.1	0.001
Tunnel diameter (mm)	2.7 ± 0.9	3.2 ± 1.0	0.002
PFO closure performed, n (%)	102 (39.2%)	34 (60.7%)	0.004
Glucose (mg/dL)	102 (92–118)	110 (96–136)	0.041
Total cholesterol (mg/dL)	192 ± 39	211 ± 43	0.002
LDL (mg/dL)	118 ± 33	132 ± 36	0.010
HDL (mg/dL)	46 ± 11	41 ± 10	0.003
Triglyceride (mg/dL)	146 (104–192)	172 (121–226)	0.012
ROPE score	6.4 ± 1.7	5.8 ± 1.6	0.018
Non-HDL (mg/dL)	146 ± 37	170 ± 40	<0.001
Non-HDL-C/HDL-C ratio	2.86 (2.30–3.45)	3.56 (2.82–4.42)	<0.001
Creatinine (mg/dL)	0.89 ± 0.18	0.97 ± 0.22	0.006

**Abbreviations:** COPD: Chronic Obstructive Pulmonary Disease, PFO: Patent Foramen Ovale, C: cholesterol, LDL: Low-Density Lipoprotein, HDL: High-Density Lipoprotein, ROPE: Risk of Paradoxical Embolism.

**Table 2:** Multivariable logistic regression analysis for prediction of cryptogenic stroke.

Variable	OR	95% CI	*p*
PFO tunnel length (mm)	1.096	1.003–1.196	0.042
PFO tunnel diameter (mm)	1.425	1.063–1.911	0.018
Glucose (mg/dL)	1.016	1.002–1.031	0.030
Non-HDL-C/HDL-C ratio	1.881	1.310–2.700	<0.001
ROPE score	1.848	1.490–2.295	<0.001

**Abbreviations**: PFO: Patent Foramen Ovale, ROPE: Risk of Paradoxical Embolism, C: cholesterol, HDL: High-Density Lipoprotein.

**Table 3:** Multivariable logistic regression analysis according to quartiles of the non-HDL-C/HDL-C ratio.

Quartile		Adjusted OR *	95% CI	*p*
Q1	Reference	-	-	-
Q2 vs. Q1		3.95	1.13–17.13	0.033
Q3 vs. Q1		5.95	1.75–25.63	0.004
Q4 vs. Q1		9.41	2.77–40.79	<0.001
*p* for trend				<0.001

* Odds ratios were adjusted for ROPE score, glucose level, PFO tunnel length, and PFO tunnel diameter using multivariable logistic regression.

## Data Availability

The data presented in this study are available on request from the corresponding author due to privacy.

## References

[1] Mojadidi M.K., Zaman M.O., Elgendy I.Y., Mahmoud A.N., Patel N.K., Agarwal N., Tobis J.M., Meier B. (2018). Cryptogenic Stroke and Patent Foramen Ovale. J. Am. Coll. Cardiol..

[2] Kleindorfer D.O., Towfighi A., Chaturvedi S., Cockroft K.M., Gutierrez J., Lombardi-Hill D., Kamel H., Kernan W.N., Kittner S.J., Leira E.C. (2021). 2021 Guideline for the Prevention of Stroke in Patients with Stroke and Transient Ischemic Attack. Stroke.

[3] Sposato L.A., Albin C.S., Elkind M.S., Kamel H., Saver J.L. (2024). Patent Foramen Ovale Management for Secondary Stroke Prevention: State-of-the-Art Appraisal of Current Evidence. Stroke.

[4] Kent D.M., Ruthazer R., Weimar C., Mas J.L., Serena J., Homma S., Di Angelantonio E., Di Tullio M.R., Lutz J.S., Elkind M.S. (2013). An index to identify stroke-related vs incidental patent foramen ovale in cryptogenic stroke. Neurology.

[5] Kent D.M., Wang A.Y. (2025). Patent Foramen Ovale and Stroke: A Review. JAMA.

[6] Hansen M.K., Mortensen M.B., Olesen K.K.W., Thrane P.G., Maeng M. (2024). Non-HDL cholesterol and residual risk of cardiovascular events in patients with ischemic heart disease and well-controlled LDL cholesterol: A cohort study. Lancet Reg. Health Eur..

[7] Liu Y., Jin X., Fu K., Li J., Xue W., Tian L., Teng W. (2023). Non-traditional lipid profiles and the risk of stroke: A systematic review and meta-analysis. Nutr. Metab. Cardiovasc. Dis..

[8] Liu Z., Lin X., Zeng L., Zhang H., Guo W., Lu Q., Huang C., Wang J., Liu P., Chang Q. (2023). Elevated non-HDL-C/HDL-C ratio increases the 1-year risk of recurrent stroke in elderly patients. BMC Geriatr..

[9] Kim H., Kim J.T., Lee J.S., Kim B.J., Kang J., Lee K.J., Park J.M., Kang K., Lee S.J., Kim J.G. (2024). Impact of non-traditional lipid profiles on 1-year vascular outcomes in ischemic stroke patients with prior statin therapy and LDL-C <100 mg/dL. Sci. Rep..

[10] Alsheikh-Ali A.A., Thaler D.E., Kent D.M. (2009). Patent foramen ovale in cryptogenic stroke: Incidental or pathogenic?. Stroke.

[11] Kent D.M., Saver J.L., Ruthazer R., Furlan A.J., Reisman M., Carroll J.D., Smalling R.W., Jüni P., Mattle H.P., Meier B. (2020). Risk of Paradoxical Embolism (RoPE)-Estimated Attributable Fraction Correlates with the Benefit of Patent Foramen Ovale Closure: An Analysis of 3 Trials. Stroke.

[12] Sniderman A.D., Williams K., Contois J.H., Monroe H.M., McQueen M.J., de Graaf J., Furberg C.D. (2011). A Meta-Analysis of Low-Density Lipoprotein Cholesterol, Non–High-Density Lipoprotein Cholesterol, and Apolipoprotein B as Markers of Cardiovascular Risk. Circ. Cardiovasc. Qual. Outcomes.

[13] Libby P. (2002). Inflammation in atherosclerosis. Nature.

[14] Barter P.J., Nicholls S., Rye K.A., Anantharamaiah G.M., Navab M., Fogelman A.M. (2004). Antiinflammatory properties of HDL. Circ. Res..

[15] Mineo C., Shaul P.W. (2012). Novel biological functions of high-density lipoprotein cholesterol. Circ. Res..

[16] Badimon L., Vilahur G. (2014). Thrombosis formation on atherosclerotic lesions and plaque rupture. J. Intern. Med..

[17] Varbo A., Nordestgaard B.G. (2014). Remnant cholesterol and ischemic heart disease. Curr. Opin. Lipidol..

[18] Turc G., Calvet D., Guérin P., Sroussi M., Chatellier G., Mas J.L., CLOSE Investigators (2018). Closure, Anticoagulation, or Antiplatelet Therapy for Cryptogenic Stroke with Patent Foramen Ovale: Systematic Review of Randomized Trials, Sequential Meta-Analysis, and New Insights From the CLOSE Study. J. Am. Heart Assoc..

[19] Wessler B.S., Thaler D.E., Ruthazer R., Weimar C., Di Tullio M.R., Elkind M.S., Homma S., Lutz J.S., Mas J.L., Mattle H.P. (2014). Transesophageal echocardiography in cryptogenic stroke and patent foramen ovale: Analysis of putative high-risk features from the risk of paradoxical embolism database. Circ. Cardiovasc. Imaging.

[20] Piovani D., Sokou R., Tsantes A.G., Vitello A.S., Bonovas S. (2023). Optimizing Clinical Decision Making with Decision Curve Analysis: Insights for Clinical Investigators. Healthcare.

[21] Pencina M.J., D’Agostino R.B., D’Agostino R.B., Vasan R.S. (2008). Evaluating the added predictive ability of a new marker: From area under the ROC curve to reclassification and beyond. Stat. Med..

[22] Caso V., Turc G., Abdul-Rahim A.H., Castro P., Hussain S., Lal A., Mattle H., Korompoki E., Søndergaard L., Toni D. (2024). European Stroke Organisation (ESO) Guidelines on the diagnosis and management of patent foramen ovale (PFO) after stroke. Eur. Stroke J..

